# Are older and seriously ill inpatients planning ahead for future medical care?

**DOI:** 10.1186/s12877-019-1211-2

**Published:** 2019-08-05

**Authors:** Amy Waller, Rob Sanson-Fisher, Balakrishnan R (Kichu) Nair, Tiffany Evans

**Affiliations:** 1 Health Behaviour Research Collaborative, School of Medicine and Public Health, Faculty of Health and Medicine, University of Newcastle, Callaghan, NSW 2308 Australia; 2 Hunter Medical Research Institute, New Lambton Heights, NSW 2305 Australia; 3 John Hunter Hospital, Hunter New England Local Health District, New Lambton Heights, NSW 2305 Australia; 4 School of Medicine and Public Health (Medical Education and Professional Development), University of Newcastle, Callaghan, NSW 2308 Australia; 5 Clinical Research Design and Statistics Support Unit, Hunter Medical Research Institute, New Lambton Heights, NSW 2305 Australia

**Keywords:** End-of-life, Advance care planning, Acute care

## Abstract

**Background:**

Despite the perceived ethical, personal and health service benefits of advance care planning (ACP), the extent to which older and seriously ill Australian inpatients have considered future health decisions remains uncertain. This study aimed to determine in a sample of older and seriously ill inpatients, the proportion who had: 1) engaged in four advance care planning (ACP) activities; 2) not engaged in ACP activities but wanted to; and 3) reasons why they had not engaged.

**Methods:**

Cross-sectional face-to-face standardised interview survey with inpatients in a tertiary referral centre who were either: aged 80+ years; aged 55+ years with progressive chronic disease(s); or judged by treating clinicians as having a life expectancy of less than 12 months. Patients indicated whether they had engaged in four ACP activities: (1) appointed medical substitute-decision-maker(s), (2) recorded end-of-life wishes in an advance directive or care plan; and talked about their end-of-life wishes with their: (3) support persons and/or (4) doctors. Patients who had not engaged in activities were asked whether they wished this to occur and reasons why.

**Results:**

One hundred eighty-six inpatients consented to the study (80% of approached). Of these, 9% (*n* = 16) had engaged in four ACP activities; 27% (*n* = 50) had not engaged in any. Half (*n* = 94, 52%) had appointed a medical substitute-decision-maker, 27% (n = 50) had recorded wishes in an advance directive or care plan, 51% (*n* = 90) had talked about their end-of-life wishes with support persons and 27% (*n* = 48) had talked with their doctor. Patients who wanted to, but had not, engaged in the four ACP activities were unaware they could record wishes or appoint decision-makers, or indicated providers had not initiated conversations.

**Conclusion:**

Relatively few inpatients had engaged in all four ACP activities. More inpatients had discussed end of life issues with family and appointed substitute decision makers, than completed written documents or talked with doctors. Community education and a more active role for community and hospital-based providers in supporting patients and families to collaboratively resolve end-of-life decisions may increase the probability wishes are known and followed.

**Electronic supplementary material:**

The online version of this article (10.1186/s12877-019-1211-2) contains supplementary material, which is available to authorized users.

## Background

Advance care planning (ACP) is an “ongoing process that supports adults at any age or stage of health in understanding and sharing their personal values, life goals, and preferences regarding future medical care” [1]. ACP is relevant for people considering the likely medical outcomes for treatment decisions at the end of life; and clarifying a person’s values and what their goals for future care might be [2]. ACP includes a range of key activities including conversations about values, preferences and important priorities with support persons and/or healthcare providers. ACP conversations often lead to the completion of an advance care directive, which is a written document recognised by common law or statutory legislation and/or legal appointment of someone to make decisions on their behalf (i.e. a substitute decision-maker) [3]. ACP has improved compliance with individuals’ goals of care and end-of-life wishes, reduced depressive symptoms and family stress [4, 5], and lowered costs and hospital presentations [6].

A systematic review reported that many frail and older people who would like to engage in advance care planning are not offered this opportunity [7]. Such discussions are often delayed until a person is imminently dying [8], even though an estimated 40% of people will lack capacity to participate in decisions in these circumstances. Much of the available data on ACP has been obtained in the United States of America (USA) over the past three and a half decades, with the reported prevalence of advance care directive ownership ranging from 26% to as high as 70% among those aged 65 years or over [9]. An international comparison study found that completion of written plans describing treatment options varied among 15,617 older adults in Germany (58%), Australia (31%), the United Kingdom (20%), Canada (46%) and Norway (4%) [10]. Overseas findings may not generalise to the Australian environment given differences in health care delivery and political and societal norms [2].

In comparison, there is limited patient-reported data on the level of engagement in different ACP activities in Australia [11], particularly amongst those who are older (defined here as 55 years of age or over) and those who have been diagnosed with life limiting illnesses other than cancer [11]. In the first national population-based study, only 14% of 2045 adults in the general community had prepared an advance care directive [12]. People were much more likely to have completed wills and appointed financial powers of attorney in comparison [12]. One third of 1600 older Australians in the community reported having prepared a written document outlining their end-of-life wishes in another study [10]. More recently, a prevalence audit study reported an overall rate of advance care directive ownership among 2285 people aged 65 years or over of 30%. Rates ranged from 48% among residents of aged care facilities to 16% of hospital patients and 3% of general practice patients [13].

Previous studies of ACP engagement often rely on retrospective audits or provider views [11, 13–15]. An advantage of obtaining patient views over these methods is that uptake and utility can be explored in the context of patients’ preference to engage in ACP [11]. Eliciting preferences at a particular time point can provide greater clarity about whether rates of uptake represent a gap in care or are a result of patient preference. Furthermore, very few studies have explored Australian inpatients reasons for not having engaged in ACP [16, 17]. Consequently, this study examined in a sample of older and seriously ill inpatients, the proportion who had: 1) engaged in each of four key advance care planning activities; 2) not engaged in the advance care planning activities but wanted to; and 3) their reasons why they had not engaged.

## Methods

### Design

A cross-sectional interview study of older and seriously ill inpatients recruited from general medicine, renal and geriatric wards in a single tertiary teaching hospital in Australia.

### Sample

Potentially eligible inpatients were admitted to participating wards and judged by staff as being cognitively and physically able to give informed consent and complete the interview. Criteria were adapted from a previous Canadian study [18]. Eligible patients were either: (a) aged 80 years and over; (b) aged 55 years or over and diagnosed with one of the following conditions: (i) congestive heart failure; (ii) chronic obstructive lung disease; (iii) cirrhosis; (iv) metastatic cancer; and/or (v) chronic renal failure; or (c) aged 55 years or over and judged by physicians to have a life expectancy of less than 12 months. Only those admitted for ≥48 h were approached, allowing time for necessary emergency care to be delivered [18].

### Procedure

The charge nurse in the selected wards identified eligible inpatients from ward lists. If the inpatient indicated they were willing to talk to a trained research assistant (RA), the RA then provided verbal and written information that the study was exploring peoples’ views about end-of-life care and decision-making, answered any questions the inpatient had and obtained written informed consent. Standardised interviews were conducted with consenting inpatients at their bedside. Individuals were able to complete the interview at a time that their family member was present if they preferred, however patients answered the survey items themselves. The age and sex of all eligible non-consenting inpatients was recorded.

### Development of survey

Establishing the validity and reliability of measures of end of life and advance care planning remains a difficult area. Published literature in the field highlights this problem and the pragmatic, ethical, and psychometric difficulties have been acknowledged [3, 19–21]. At the time of data collection, no psychometrically robust self-report measures were available that included the broad range of end of life topics covered in this study. Therefore, a study-specific survey has developed using the following process. First, healthcare providers and consumers participated in 20-min individual interviews to elicit their views and experiences. Potential items were then reviewed by a panel including a geriatrician, oncologist, surgeon, nurse, nephrologist, palliative care physician and behavioural scientists until consensus on content and format of items was reached. Items were then modified and pilot tested with a convenience sample of 20 inpatients for acceptability, relevance and clarity, and refined based on their feedback.

### Measures

The items described here (see Additional File 1) are drawn from a larger set of survey items, some of which will be reported elsewhere because they address different themes*.* The following definitions of ‘ACP’ were provided. “ACP provides an opportunity for people to think, discuss and plan for the medical treatment they would prefer if they became too ill in the future to express their wishes. Everyone should consider advance care planning, regardless of their age or health. But it is particularly important for people who have ongoing health problems. These questions ask for your views and experiences in talking and making decisions about your future medical care, including end of life care”.

#### Self-reported engagement in ACP activities

Patients were asked whether they had engaged in each of the four ACP activities including: (1) “*written down your wishes for end-of-life health care (e.g. in an advance directive or care plan*”); (2) “*appointed a medical substitute-decision-maker (i.e. legally appointed to make medical decisions on your behalf if you can’t yourself)*”. (3) “*talked about the type of end-of-life health care you would like to receive with your support person*”; and (4) “*talked about the type of end-of-life health care you would like to receive with your doctor*”. Standard definitions were provided. Response options for each item were ‘yes’, ‘no but I would like to’, ‘no but I do not want to’ or ‘unsure’. Further explanation was offered if these terms were not clear.

#### Reasons for non-engagement

For each item, open-ended responses were then sought by the interviewer. Those who responded to the questions with a ‘no, but I would like to’ were asked “*What has stopped you from engaging in the [ACP activity]?*”. Those who indicated that they did not want to engage in any of the listed ACP activities were asked why they did not want to engage.

#### Financial planning

Patients were asked whether they had: (1) “*made a will*”; and (2) “*appointed an enduring financial power of attorney (i.e. someone legally appointed to make financial decisions on your behalf”*). Response options for each item were ‘yes’, ‘no but I would like to’, ‘no but I do not want to’ or ‘unsure’.

#### Socio-demographic and clinical variables

Were obtained via patient self-report, including sex, age, country of birth, religion, living arrangements and perceived quality of life and health (*both rated on a single item 0–10 scale; higher scores indicated better quality of life or health*). Clinical items included self-reported reason(s) for admission and medical condition(s).

### Statistical analysis

Statistical analyses were programmed using SAS software v9.4 (SAS Institute, Cary, North Carolina, USA). Socio-demographic, clinical characteristics, and responses to ACP questions were summarised as frequencies, and percentages of non-missing responses. Open-ended responses were elicited for patients who indicated they had not engaged in ACP however not all patients chose to give a response. Chi-square or Fisher’s exact tests for independence were performed for survey items with sub-group (i.e. 55–79 years vs 80+ years). The sample size allowed estimation of population proportions within a 7% margin of error.

## Results

### Sample

A total of 186 inpatients consented to participate (80% of 233 approached). Of these, 64% were aged 80 years or over, 57% were female and 95% resided in the community either in their own home or the home of a relative or friend prior to admission (see Table 1). There were significant age differences (× 2 = 5.78, *P* < 0.05; fewer 80+ year old patients) compared with those who withheld consent to participate.

**Table 1:** Socio-demographic and clinical characteristics of the participant sample

	55 79 years(*n* = 65)	80 years or over(*n* = 116)	Total(*N* = 186)
Sex			
Male	28 (43%)	49 (42%)	80 (43%)
Female	37 (57%)	67 (58%)	106 (57%)
Australian born			
Yes	56 (86%)	104 (90%)	165 (89%)
No	9 (14%)	12 (10%)	21 (11%)
Religion			
Catholic	20 (31%)	20 (18%)	40 (22%)
Anglican	14 (22%)	31 (27%)	47 (26%)
Muslim	4 (6.2%)	10 (8.8%)	14 (7.6%)
No religion	16 (25%)	28 (25%)	45 (24%)
Other	11 (17%)	25 (22%)	38 (21%)
Living arrangements			
Home alone	29 (45%)	58 (50%)	90 (49%)
Spouse/partner	23 (35%)	29 (25%)	54 (29%)
Relative	9 (14%)	17 (15%)	26 (14%)
Nursing home	3 (4.6%)	6 (5.2%)	9 (4.9%)
Other	1 (1.5%)	5 (4.3%)	6 (3.2%)
Reason for admission*			
Fall	13 (21%)	36 (31%)	49 (27%)
Pneumonia	2 (3.2%)	5 (4.4%)	7 (3.9%)
Shortness of breath	5 (7.9%)	9 (8.0%)	14 (7.7%)
Fracture	0	4 (3.5%)	4 (2.2%)
Infection	7 (11%)	8 (7.1%)	15 (8.8%)
Other	47 (72%)	68 (60%)	115 (64%)
Medical conditions*			
Cancer	9 (14%)	15 (14%)	25 (14%)
COPD	6 (9.4%)	10 (9.0%)	17 (9.4%)
Heart failure	13 (20%)	21 (19%)	35 (19%)
Renal disease	25 (39%)	10 (9.0%)	35 (19%)
Diabetes	19 (30%)	11 (9.6%)	31 (17%)
Other	40 (63%)	80 (72%)	123 (69%)

*Multiple response items, column does not add to 100%. Column frequencies may not sum to 186 due to missing data

### Engagement in four advance care planning (ACP) activities

**Fig. 1:**
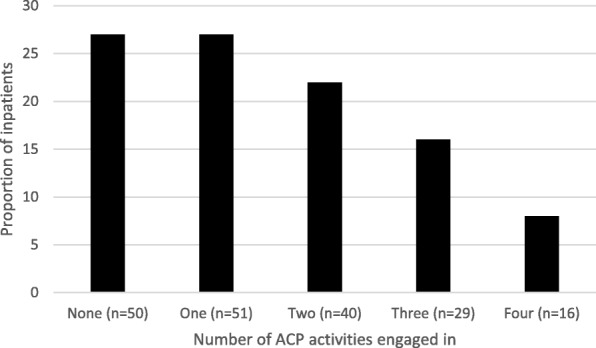
The total number of advance care planning activities engaged in by participants

Figure 1 presents the number of ACP activities patients self-reported they had engaged in (i.e. talked about their end-of-life wishes with support person(s), talked about their end-of-life wishes with doctor, recorded wishes in an advance directive or care plan and/or appointed a medical substitute-decision-maker). Overall, 9% of the respondents had (*n* = 16) engaged in all four ACP activities; 27% (*n* = 50) had not engaged in any of the identified ACP activities.

Table 2 describes the proportion of patients who had engaged in each of the four ACP activities. Overall, 51% (*n* = 90) of had talked about their end of life wishes with support persons, 52% (*n* = 94) had appointed a medical substitute-decision maker, 27% (*n* = 48) had talked about their end of life wishes with their doctor and 27% (n = 50) had written down their wishes in an advance directive or care plan. Of the 94 respondents who had appointed a medical substitute-decision-maker, the majority had appointed their children (*n* = 79) or spouse/partner (*n* = 9). However, only one third had told their healthcare providers about the appointment (32%, *n* = 29). Only 50 (27%) respondents had recorded wishes in an advance directive or care plan. Healthcare provider(s) (44%, *n* = 22), family (36%, *n* = 18) and/or solicitor(s) (18%, n = 9) were involved in the preparation of these documents. End-of-life conversations occurred most often with children (*n* = 69), spouse/partners (*n* = 20) and/or other family/friends (n = 7). End of life conversations occurred more often with general practitioners (GPs) (*n* = 34) than with hospital-based clinician(s) (*n* = 14) and/or aged care home staff (n = 3).

**Table 2:** Prevalence of engagement in advance care planning activities in whole sample and by sub-group

	Total (*N* = 186)	55–79 years (*n* = 65)	80+ years (*n* = 116)	p
Discussed preferred end of life care with doctor				0.6396
Yes	48 (27%)	14 (23%)	33 (29%)	
Not engaged but wanted to	57 (32%)	21 (34%)	35 (31%)	
Not engaged and did not want to	73 (41%)	27 (44%)	45 (40%)	
Discussed preferred end of life care with support person				0.6058
Yes	90 (51%)	28 (45%)	59 (53%)	
Not engaged but wanted to	29 (16%)	12 (19%)	17 (15%)	
Not engaged and did not want to	58 (33%)	22 (36%)	36 (32%)	
Written down wishes for end of life care				***0.0043***
Yes	50 (27%)	20 (31%)	28 (24%)	
Not engaged but wanted to	56 (31%)	27 (42%)	28 (24%)	
Not engaged and did not want to	77 (42%)	17 (27%)	59 (51%)	
Appointed a medical substitute-decision maker				0.1904
Yes	94 (52%)	31 (48%)	60 (53%)	
Not engaged but wanted to	47 (26%)	22 (34%)	25 (22%)	
Not engaged and did not want to	41 (23%)	12 (19%)	29 (25%)	

significant *p*-value is in bold and italics

### Desire to engage in each of the four ACP activities

Of the 133 patients who had not already recorded their end-of-life wishes in an advance directive or care plan, 42% (*n* = 56) wanted to do so (Table 2). Of the 88 patients who had not already appointed a medical substitute-decision-maker, 53% (*n* = 47) indicated they wish to take such an action. Further, 33% (*n* = 29/87 inpatients) who had not talked about their end-of-life wishes with their support person wanted to have such a discussion; and 44% (*n* = 57/130 inpatients) who had not talked about their end of life wishes with their doctor wanted to do this. Among participants who had not written down their wishes for end of life care, those who were aged 80+ years were less likely to want to do so compared to those aged 55–79 years with progressive diseases (51% vs 27%, *p* < 0.05). There were no significant differences between the two age groups for any other ACP activity (see Table 2).

### Reasons why patients had not engaged in ACP

Only 59% (*n* = 106) of respondents were familiar with the concept of a ‘medical substitute-decision-maker’ and 24% (*n* = 43) with an ‘advance care directive’. Among those patients who had not engaged in at least one of the four ACP activities but wanted to (*n* = 98), the most common reasons were: they had ‘just not thought about it before’ (*n* = 60); they had never heard of the activity (*n* = 31); or had not had an opportunity to engage in the activity (*n* = 6). Some inpatients reported they had initiated but had not completed the activity (e.g. made an appointment to prepare a written document) (*n* = 15). Others did not provide reasons.

For those who had not engaged in at least one of the four ACP activities but did not want to (*n* = 87), the most common reasons were they: felt their family (or doctor) knew what they wanted already (*n* = 64); were ‘*just not interested’* (*n* = 57); perceived they would not get any benefit by participating (*n* = 34); did not have a regular or trusted healthcare provider (*n* = 9); wanted to avoid burdening others (n = 6); or did not want others making decisions for them (n = 6).

### Engagement in financial planning

The majority of respondents had made a will (90%, *n* = 151), and most had an enduring financial power of attorney (74%, *n* = 124). Of those who had not already done so, 47% (n = 8 of 17) wanted to prepare a will and 51% (*n* = 19 of 37) wanted to appoint an enduring financial power of attorney.

## Discussion

Despite their estimated limited life expectancy, 27% of patients had not engaged in any of the four ACP activities and 9% had engaged in all four activities. It is acknowledged that discussion and documentation may not cover all eventualities that an individual may face regarding their future health care [22]. However, it is likely that the presence and discussion of such issues may assist patients and their substitute decision-makers so that care consistent with patient’s wishes is more likely to be provided [22].

Half of respondents had formally appointed a medical substitute-decision-maker. Without a clear designation of authority it is possible that conflicts may emerge in families and healthcare teams when there is an attempt to determine what end-of-life care an individual might prefer [23]. While the order of priority in which people are approached if someone has not been formally nominated is determined by statutes in Australia, studies show that a person’s preference does not always reflect this hierarchy. For instance, 30% of ICU patients preferred another trusted advocate rather than spouse to be their decision-maker [24]. Similar rates of documentation of end of life wishes were reported for the two groups (24 and 31%). Older age has been previously associated with greater ACP engagement [25–27]; while others have not reported any association between age and engagement [28]. Our findings suggest that younger patients with progressive diseases are an important target for initiating formal advance care planning processes, as a higher proportion indicated they had not, but wanted to write down their wishes.

While half of the respondents had discussed their end-of-life wishes with a support person, only a quarter had done so with a healthcare provider. Early conversations can increase the likelihood that patients have sufficient capacity to meaningfully engage, reduce time pressures that can prevent the patient from adequately reflecting on and discussing wishes, and enhance opportunities for multi-disciplinary input [29, 30]. Involving substitute-decision makers early may also avoid placing these individuals in an emotionally vulnerable and fraught decision situation. Of those who had talked to a healthcare provider, most reported conversations with their GP rather than a hospital-based clinician. This is not surprising, as GPs are a frequent point of contact and have a key longitudinal role in caring for older people [31]. However, not all GPs are skilled and comfortable discussing likely future scenarios for the end of life [32]. Further, GPs are unlikely to be involved in end-of-life decisions and actions for the significant proportion of older people who will die in hospital. A mechanism by which patient wishes can be effectively and accurately communicated between GPs and hospital providers is therefore important.

This is one of the first Australian studies to provide patient-reported data to quantify the gap between what older and seriously ill inpatients want and what is currently occurring in relation to ACP. Awareness of and opportunities to engage in ACP activities in the inpatient setting appear limited. To achieve improvements in ACP uptake and utility, high-quality educational strategies that clearly articulate the supporting regulatory framework should be employed with inpatients, families and providers [33]. This may help reduce uncertainty about the purpose of ACP, as well as what constitutes binding written documentation and the rights and responsibilities of substitute-decision-makers [34]. As a person’s preference may change with health or personal circumstances, providing opportunities to prepare or modify existing documentation at regular intervals can help address concerns about not being able to amend documents; or the reluctance of providers to implement documents made some time ago or where instructions are vague [35].

Not all respondents wanted to engage in ACP. Many believed families and healthcare providers already knew what they wanted with respect to end-of-life care and would make the appropriate decision when the time came [36]. This is despite these same respondents reporting that they had not discussed their end-of-life wishes with these individuals. Relying on substitute-decision-maker or provider views does not always guarantee that patients’ true preferences will be achieved, especially when conversations have not occurred [8, 18]. Consistent with previous studies [36, 37], patients also voiced concerns about the potential burden on loved ones and perceived irrelevance of ACP to their circumstances.

### Implications and future research

ACP activities are associated with better quality of life and improved compliance with end of life wishes for patients, fewer depressive symptoms and less stress in family members; [4, 5, 38] and lower costs and fewer hospital presentations [6]. Despite these potential benefits and their estimated limited life expectancy, few inpatients have engaged in all four identified ACP activities. To increase the probability that a person’s actual end-of-life wishes are known and followed, the following steps are required. Firstly, there is a need to improve end-of-life communication between patients, their families, and hospital-based providers. Information conveyed during end of life conversations with GPs is rarely available at the time end-of-life decisions are being made in hospital. Secondly, end-of-life conversations should involve the patient, substitute-decision-maker; and the most senior clinician responsible for leading and coordinating the patient’s care, given heightened emotions surrounding topics being discussed [39]. Furthermore, end-of-life wishes and details of designated substitute-decision-makers should be documented in written plans, with mechanisms in place to ensure that these are readily available to all teams involved in the patient’s care [39].

### Strengths and limitations

This study provides some of the first patient-reported data about the preferences and experiences of a large group of Australian inpatients with respect to four informal and formal ACP activities. However, there are a number of limitations. While the eligibility criteria reflect a group for whom ACP is particularly relevant, it is a single institution sample which may not be representative of results from other organisations. Replicating the survey across organisations would provide important information of use to clinicians to guide ACP and should be explored [13]. Our sample was primarily Australian born and community-dwelling. Recent Australian data also reported differences in rates of advance care directive ownership across general practice, hospital and residential aged care settings [13]. Surveying patients within each of these settings would help clarify reasons why this disparity may be occurring, including whether it reflects patient preference or represents a gap in care. The cross-sectional nature of the data is also a limitation. Prospective studies that examine people’s preferences and experiences over time and the potential impact of their choices on patient and family outcomes are needed.

## Conclusions

Few inpatients in this study had engaged in all four advance care planning (ACP) activities. Fewer inpatients had talked about their wishes for end-of-life care with doctors or recorded their wishes in an advance directive, when compared to having talked about their wishes with their family and appointing a medical substitute decision-makers. Despite an expressed desire to engage in these activities, significant barriers to ACP activities remain. To increase the probability that people’s end-of-life wishes are known and followed, education of the general community about the purpose and potential benefits of ACP is needed. Education and training of community and hospital-based providers is also required to ensure they are able to take a more active role in discussing and enacting end-of-life decisions with patients and their families.

## Additional files

Additional file 1: Standardised Interview Survey items. This lists the interview survey items completed by participants. (DOCX 18 kb)

## Data Availability

Datasets are available from the corresponding author on reasonable request.

## Abbreviations

ACD: advance care directive
ACP: advance care planning
GP: general practitioner
USA: United States of America
