# Supplementary material for: Are older and seriously ill inpatients planning ahead for future medical care?
Source: BMC Geriatr. 2019 Aug 5;19:212. doi: 10.1186/s12877-019-1211-2 (PMC6683455; doi:10.1186/s12877-019-1211-2)
Supplement: Supplementary file 1 — Standardised Interview Survey items. This lists the interview survey items completed by participants. (DOCX 18 kb) [file 12877_2019_1211_MOESM1_ESM.docx]

**Interview Survey Items**

**SECTION A: ABOUT YOU**

**1. Are you male or female?**

- Male
- Female

**2. What is your date of birth? ___ ___ / ___ ___ / ___ ___ ___ ___**

**Day Month Year**

**3. What country were you born in?**

- Australia
- Other (please specify) __________________

**4. Are you of Aboriginal or Torres Strait Islander origin?**

- 1 No
- 2 Yes, Aboriginal
- 3 Yes, Torres Strait Islander
- 4 Yes, both Aboriginal and Torres Strait Islander

**5. What is your religion (if any)?**

- Catholic
- Anglican
- Muslim
- Buddhist
- No religion
- Other (please specify) **____________________________**

**6. What are you current living arrangements?**

- At home alone
- At home with spouse/partner
- Relative’s home
- Nursing home
- Other (please specify) ____________________________

**7. Are there other people that provide you with practical and emotional support?**

**(Circle all that apply)**

- Spouse/partner
- Brother/sister
- Child(ren)
- Friends/neighbours
- Other (please specify)

**8. Does this person / they live close to you?**

- Yes
- No

**9. How would you rate your overall quality of life?**

1 2 3 4 5 6 7 8 9 10

Poor Excellent

**10. How would you rate your overall health?**

1 2 3 4 5 6 7 8 9 10

Poor Excellent

**11. Reason for admission (e.g. fall, pneumonia, fracture)**

**12. Medical conditions**

- Cancer
- COPD
- Heart failure
- Kidney/renal disease
- Other (please specify)

**SECTION B**

***Advance care planning (ACP) provides an opportunity for people to think, discuss and plan for the medical treatment they would prefer if they became too ill in the future to express their wishes. Everyone should consider advance care planning, regardless of their age or health. But, it is particularly important for people who have ongoing health problems. These questions have nothing to do with your current state of health. We are asking everyone admitted to the hospital to answer these questions.***

**13. Do you have a will (i.e. written document that describes who will receive your assets when you die)? (For each response option, follow-up questions were asked)**

- Yes
- No but I would like to
- No but I did not want to
- Unsure

**14. Do you know what an enduring financial power of attorney is (i.e. someone legally appointed to make financial decisions on your behalf if you can’t yourself)?**

- Yes (ask for definition)
- No
- Unsure

**15. Have you appointed an enduring financial power of attorney (i.e. someone legally appointed to make medical decisions on your behalf if you can’t yourself)?**

- Yes
- No but I would like to
- No but I do not want to
- Unsure

**16. Do you know what an Advance Directive is?**

- Yes (ask for definition)
- No
- Unsure

**17. Have you written down your wishes for end of life care (e.g. in an advance directive or care plan – a written document that describes the type of medical care you would want or not want e.g. CPR, breathing machine)?**

- Yes
- No but I would like to
- No but I do not want to
- Unsure

**18. Do you know what an enduring guardian is?**

- Yes (ask for definition)
- No
- Unsure

**19. Have you appointed an enduring guardian (i.e. someone legally appointed to make medical decisions on your behalf if you can’t yourself)**

- Yes
- No but I would like to
- No but I do not want to
- Unsure

**20. Have you talked about the type of end of life care you would like to receive with your support person / enduring guardian:**

- Yes
- No but I would like to
- No but I do not want to
- Unsure

**21. Have you talked with your health care team about the type of end of life care you would like to receive:**

- Yes
- No but I would like to
- No but I do not want to
- Unsure

**SECTION B: (Data not presented here)**

**23. If you were unable to make decisions on your own, would you prefer your end of life care to be decided by:**

- A care plan you had made before you got too sick to make decisions
- Your doctor with your family/friends, based on their views of what was best
- Only your doctor, based on their view of what was best for you
- Only your family/friends, based on their view of what was best for you

**23. Is there anyone you would not want to make decisions on your behalf?**

- Yes
- No
- Unsure
- Prefer not to answer

**24. If you were able to choose, where would you most prefer to be cared for at the end of life? (Response options follow-up buy asking reasons)**

- In your own home
- In a hospital
- In a hospice / palliative care unit
- In a nursing home

**25. If you were able to choose, where would you most prefer to die? (Response options follow-up buy asking reasons)**

- In your own home
- In a hospital
- In a hospice / palliative care unit
- In a nursing home

**26. If you could choose, would you prefer care that focuses on (select one):**

- Extending life as much as possible, even if it meant more pain and discomfort
- Relieving pain and discomfort as much as possible, even if it meant not living as long
- Unsure

**27. Has anyone talked to you about your life expectancy?**

- Yes
- No but I would like to
- No but I did not want to
- Unsure

**If you needed end of life care, would you want:**

***28. The option of being sedated if the emotional or physical pain became too much***

- Strongly disagree
- Disagree
- Unsure
- Agree
- Strongly agree

***29. To* be *able to access medications that allow you to end your own life if it is your wish***

- Strongly disagree
- Disagree
- Unsure
- Agree
- Strongly agree
